# Systematic Study of Steroid Drugs’ Ability to Cross Biomembranes—The Possible Environmental Impact and Health Risks Associated with Exposure During Pregnancy

**DOI:** 10.3390/membranes15010004

**Published:** 2024-12-26

**Authors:** Anna W. Sobańska, Aleksandra Orlikowska, Karolina Famulska, Lovro Bošnjak, Domagoj Bosiljevac, Aleksandra Rasztawicka, Andrzej M. Sobański

**Affiliations:** 1 Department of Analytical Chemistry, Faculty of Pharmacy, Medical University of Lodz, 90-151 Lodz, Poland; 2 Faculty of Pharmacy, Medical University of Lodz, 90-151 Lodz, Poland; alicja.orlikowska@student.umed.lodz.pl (A.O.); karolina.famulska@student.umed.lodz.pl (K.F.); aleksandra.rasztawicka@student.umed.lodz.pl (A.R.); 3 Faculty of Pharmacy and Biochemistry, University of Zagrzeb, 10000 Zagreb, Croatia; lovro.bosnjak1745@gmail.com (L.B.); domagoj.bosiljevac0@gmail.com (D.B.); 4 Faculty of Chemistry, University of Lodz, 91-403 Lodz, Poland; andrzej.sobanski@edu.uni.lodz.pl

**Keywords:** biomembranes, permeability, steroids, gestation

## Abstract

Thirty-seven steroid drugs of different types were investigated in silico for their environmental and pharmacokinetic properties (partition between soil and water, bioaccumulation in aquatic organisms, ability to be absorbed from the gastrointestinal tract and to cross biological barriers—skin, blood–brain barrier and placenta) using on-line tools and novel QSAR models. The same drugs were studied by Molecular Docking in the context of their ability to interact with two enzymes—glutathione S-transferase (GST) and human N-acetyltransferase 2 (NAT2), which are involved in the placenta’s protective system against harmful xenobiotics. Steroid drugs are released to the environment from households, hospitals, manufacturing plants and farms (e.g., with natural fertilizers) and they can affect the aquatic life (reproduction and development of aquatic organisms), even at sub-ng/L concentrations. It was established that the majority of studied drugs are mobile in soil, so they may reach surface waters far from point of discharge, e.g., from farming; however, only a few of them are likely to bioaccumulate. All of them can be absorbed orally or through skin, and they are also expected to cross the placenta. Over 30% of studied compounds are likely to pass through the blood–brain barrier (although five compounds in this group are likely P-gp substrates, which may reduce their activity in the central nervous systems); they have also very high affinity for both studied enzymes.

## 1. Introduction

Research on environmental exposure to steroid drugs has generated a lot of interest in recent times. Steroids are organic compounds built of the carbon skeleton, consisting of four fused rings: three cyclohexane rings and one cyclopentane ring [1]. These compounds belong to several classes: adrenal cortical hormones (glycocorticosteroids, mineralocorticosteroids) and sex hormones (androgens, estrogens, progestogens) [1]. Research indicates that the steroids mainly responsible for polluting the environment are hormonal agents, especially estrogens. Among natural steroids, estrone and estradiol are prominent, while ethinylestradiol is a key synthetic steroid [2]. These types of pollutants can have a significant impact on disrupting the functions of the endocrine system. The metabolism of estrogens proceeds in the liver and kidneys by conjugation of estriol, epiestriols, and catechol estrogens to glucuronides and sulfates, which are highly water-soluble and are rapidly excreted by kidneys [3]. In this way, steroids can reach the natural environment via sewage. Another source of steroids’ exposure among unaware individuals is by supplement consumption. For example, recent studies have shown that dietary supplements used in rheumatic conditions has resulted in the emergence of products adulterated with steroids, mainly corticosteroids, causing numerous potential side effects: psychosis, anxiety, hyperglycemia, and muscle atrophy [4].

Although there are many reports on the occurrence of pharmaceutical residues in the ecosystem, there is still lack of detailed information about the effects of exposure on human health, especially in childbearing; little is known on how contamination with steroid drugs effects the fetus. Steroids, especially synthetic substances, are commonly used in pharmacological treatment during pregnancy, for example to reduce the risk of miscarriage (progesterone), in prevention and treatment of obstetric conditions (progestogens) and to optimize the health of the newborn (antenatal corticosteroids) [5]. The aim of this research is to give a systematic overview of environmental and pharmacokinetic properties of 37 selected steroid drugs from different families (taken from [6])—with particular focus on those features that may affect an unborn child.

## 2. Materials and Methods

### 2.1. Calculated Pharmacokinetic Parameters

Bioconcentration factors (log ***BCF***) and soil/water partition coefficients (log ***K***_oc_) were calculated using EpiSuite package v. 4.11, US Environmental Protection Agency, Washington, DC, USA [7,8]. Skin permeability coefficients (log ***K***_p_), human intestinal absorption (***HIA***) and the parameters related to the blood–brain barrier permeability (log ***BB***, log ***PS***) were calculated using pkCSM software [9]. Affinity of steroids for P-gp was assessed qualitatively using SwissADME software [10]. Pharmacokinetic and environmental properties of studied steroids are given in Table 1.

### 2.2. Molecular Descriptors

Mordred descriptors [11] used to compute steroids’ fetus-to-mother blood concentration ratio (log ***FM***) were calculated using the OChem online platform (https://ochem.eu/home/show.do accessed on 10 November 2024). Molecular descriptors investigated in the context of log ***K***_p_,_uu,br_ predictions and ligand–protein binding studies were calculated using SwissADME [10] and pkCSM software [9] (Supplementary Materials). Logarithmic ***MDKC*** and ***PAMPA*** permeabilities (cm/s) were calculated with ADMETlab3.0 [12].

### 2.3. Multiple Linear Regression (MLR) Model of Log **K**_p,uu,br_

Models of log ***K***_p,uu,br_ were generated using Statistica v. 13.3 (StatSoft Kraków, Kraków, Poland) by Multiple Linear Regression in forward stepwise mode, using molecular descriptors calculated by SwissADME (Supplementary Materials) and two computational permeability descriptors (***MDKC*** and ***PAMPA***). The set of 74 compounds whose ***K***_p,uu,br_ are known [13] was divided randomly onto two subsets: a training set of 60 compounds and a test set of 14 compounds. Pre-selection of descriptors to avoid multicollinearity was manual, based on a tolerance matrix (Supplementary Materials). Tolerances were calculated as (1–R^2^) and it was assumed that two descriptors are colinear if the tolerance value between them is <0.1 [14]. Models were validated using (i) RMSEP_ext_—Root Mean Square Error of Prediction for the external test set; and (ii) RMSECV—Root Mean Square Error of internal, 5-fold Cross-Validation (with randomly ordered compounds ***1*** to ***60*** divided into 5 subsets: ***1*** to ***12***, ***13*** to ***24***, ***25*** to ***36***, ***37*** to ***48*** and ***49*** to ***60***) [15].

### 2.4. Molecular Docking Studies

Molecular docking was carried out using the SwissDock package (http://www.swissadme.ch/, accesses on 20 October 2024), AutoDock Vina mode [16,17], using crystal structures of GST and NAT2 proteins taken from the RCSB Protein Data Bank (https://www.rcsb.org; PDB ID’s: 1LJR and 2PFR, respectively, accesses on 20 October 2024). The binding sites and settings for both proteins were taken from [18] (identified based on GST and NAT2 complexes with glutathione and coenzyme A, respectively), and they are as follows:

- 2PFR: sampling exhaustivity: 8, box size: x = 24.365, y = 32.644, z = 24.861, box center: x = 8.326, y = 38.309, z = 65.486
- 1LJR: sampling exhaustivity: 8; box size: x = 22.139; y = 29.221; z = 33.415; box center: x = 12.398; y = 67.11; z = 2.305

Affinities of studied steroids for both proteins ***k*** (kcal/mol) were recorded.

## 3. Results and Discussion

### 3.1. Mobility in the Soil–Water Compartment

Occurrence of steroid drugs in soil and water resources and their mobility in the soil–water compartment are serious concerns [19]. As with many other pharmaceuticals, steroids are likely to be released to the soil and surface waters from households, hospitals and production facilities—and from agriculture, as some of them are administered to farm animals and reach the soil with natural fertilizers [20]. Steroid hormones, both natural and synthetic, influence many physiological processes in all vertebrates. They are present in surface waters all over the world, and their presence, even at concentrations lower than 0.1 ng/L, may adversely affect reproduction, impair the development of offspring, suppress somatic growth and delay breeding age [21]. Steroids exhibit very strong synergistic effects—when present in mixtures, they affect physiological processes even at concentrations far below the reported levels of activity for individual compounds [22].

The mobility of chemicals in soil causes them to travel long distances from the point of discharge, and the main parameter related to the mobility of compounds in soil is the soil–water partition coefficient ***K***_oc_ (normalized to the soil organic-carbon content to reduce the differences among soils) [23]. Although log ***K***_oc_ can be measured directly, high-throughput chromatographic or computational methods of log ***K***_oc_ determination have been developed [24,25,26,27]. In this study, log ***K***_oc_ was calculated according to two well-established models [8]:

log ***K_oc_***^(1)^ = 0.52 ***MCI*** + 0.60 + corrections (1)

log ***K_oc_***^(2)^ = 0.55 log ***K_o/w_*** + 0.93 + corrections (2)

where ***MCI***—first order molecular connectivity index, log ***K_o/w_***—estimated octanol–water partition coefficient (***KOWWIN***) [7], and the value given in Table 1 is the average of values calculated according to Equations (1) and (2).

One of the most widely accepted models of log ***K***_oc_ (Equation (2)) is based on the estimated octanol–water partition coefficient of compounds. The application of computed rather than experimental values of octanol–water partition coefficients in models of pharmacokinetic or environmental properties is a common practice, considering the poor availability of experimental data for a great majority of drug candidates or environmental pollutants. Luckily, several in silico or chromatography-based methods of lipophilicity estimation have been developed and validated against experimental data, including also steroid compounds [28,29,30,31].

According to US EPA, compounds may belong to one of six soil mobility classes, as shown in Table 2 (similarly, McCall assigns compounds to six classes, but the cut-off values are slightly different) [32]—and, according to this classification, only a few studied compounds can be considered immobile or hardly mobile in soil; the majority of steroid drugs investigated in this study are at least moderately mobile. However, we should not ignore the fact that log ***K***_oc_ might not contain enough information to function as a single indicator of the mobility of chemicals [33], especially in the context of drinking-water safety.

### 3.2. Bioconcentration and Bioaccumulation in Aquatic Organisms

The *BCF* (bioconcentration factor) of solutes in aquatic organisms is an important parameter, because many xenobiotics are released to the ecosystem; once they have become environmental pollutants, they can be absorbed by unaware individuals, for example via contaminated water or food. The fish bioconcentration factor (***BCF***) is the ratio of the concentrations of a compound in the organism and water in the state of equilibrium, accounting for the absorption via the respiratory route (e.g., gills) and skin; the bioaccumulation factor (***BAF***) is a similar parameter, but it accounts also for dietary absorption. It is assumed that compounds that bioaccumulate have a ***BCF*** or ***BAF*** > 5000 or 2000 (depending on the source) [34]. If no ***BCF*** or ***BAF*** data are known, the octanol-water partition coefficient (log ***K***_o/w_, log ***P***) is used as a criterion of bioaccumulation, with the assumed cut-off values separating non-bioaccumulating and bioaccumulating compounds between 3.3 and 5 [34,35,36,37]. This approach, however, does not account for the fact that highly-lipophilic molecules (log ***K***_o/w_ > ca. 7) do not bioconcentrate as readily as expected, based solely on their lipophilicity [38,39].

Experimental ***BCF*** or ***BAF*** values have been measured for several hundred of compounds, but for the majority of organic environmental contaminants such data do not exist, and their ***BCF*** or ***BAF*** values need to be determined in silico. In this study, the log ***BCF*** values for 37 steroids were calculated according to US EPA [7]. It was established, based on the cut-off value ***BCF*** = 2000 given above, that studied steroids are unlikely to be bioaccumulative—with the exceptions of testosterone enanthate, hydrocortisone octanoate and estradiol benzoate.

### 3.3. Skin Permeability

Steroid drugs are used topically, depending on the class, e.g., as contraceptives or in hormone replacement therapy [40,41], in treatment of different skin ailments [42] and in sport doping [43]—with up to 10% of applied doses being absorbed, depending on the delivery system [41]; the transdermally absorbed steroids are, in particular, androgens, estrogens and progestins. The skin permeability of steroids has been a subject of interest for a relatively long time [6,44]. In this study, the skin permeability coefficient (log ***K***_p_) was calculated for 37 steroid drugs, using the pkCMS platform. Relatively high values were obtained for studied compounds—this being the reason why so many of them (including e.g., progesterone) can be administered transdermally [40,45], but also facilitating incidental exposure.

### 3.4. Human Intestinal Absorption

In this study Human Intestinal Absorption (%) was calculated using the pkCMS platform; none of the studied steroids is poorly absorbed from the gastrointestinal (GI) tract, which is in line with their drug-likeness, according to Lipinski’s Rule of 5 [46,47] (a crude filter that makes it possible to pick orally available drugs using a few simple molecular properties: ***MW*** ≤ 500, calculated octanol-water partition coefficients expressed as ***cLogP*** ≤ 5 (or ***MLOGP*** ≤ 4.15), H-bond acceptor and donor counts ***nHA***≤ 10, ***nHD*** ≤ 5). There is no more than one violation, as established using the SwissADME platform [10].

### 3.5. Placenta Permeability

The placenta permeability of studied steroids is expressed as their log ***FM*** (fetus-to-mother blood concentration in the state of equilibrium), calculated according to Equation (3), developed earlier [48], and based on a set of 54 compounds whose experimental log ***FM*** values are available [49].

log ***FM*** = −0.038 (± 0.162) − 0.0081 (± 0.0025) ***ZMIC1*** − 0.011 (± 0.002) ***EState_VSA8*** + 0.20 (± 0.08) ***GATS7Z*** − 0.12 (± 0.06) ***Lipinski*** (3)

(n = 40, R^2^ = 0.80, R^2^_adj._ = 0.78, F = 35.9, *p* < 0.01, RMSEP_ext_ = 0.20).

According to some previous studies [50], the majority of small molecules of low-to-medium lipophilicity are absorbed across the placenta by passive diffusion and the often-assumed cut-off value between compounds whose transport across the placenta is easy and those that do not cross the placenta easily is log ***FM*** = −0.52 [50]. In the group of studied steroids there are a few that, formally, belong to the low-placenta-permeability group (Table 1), but their log ***FM*** values are relatively near the threshold value, so (assuming that the placenta permeability is a certain continuum) they must not be regarded as absolutely safe for a fetus.

### 3.6. Blood–Brain Barrier Permeability

The blood–brain barrier (BBB) separates brain tissues and blood, both physically and enzymatically. Compounds’ ability to cross this barrier is expressed by different parameters, of which the most significant are the following:

(i) ***BB*** (defined as the ratio of the total drug concentration in the brain to that in blood in the state of equilibrium) [51].

(ii) ***PS*** in the unit [mL/min/g brain] and obtained via in situ brain perfusion studies using the Renkin–Crone equation: ***PS*** = −***F***∙ln (1 − ***K***_in_/***F***), where ***F*** is the cerebral blood or perfusion flow rate, ***K****_in_* = (***Q***_br_/***Q***_pf_)/***T*** is the unidirectional transfer constant, ***Q****_br_* is the concentration, corrected for the vascular volume, of compound in the brain, ***Q****_pf_* is the concentration of a compound in the perfusion fluid and ***T*** is the perfusion time [52].

(iii) The unbound brain-to-plasma concentration ratio (***K***_p,uu,brain_) or the unbound CSF (cerebrospinal fluid-to-plasma concentration ratio (***K***_p,uu,CSF_) [13,53,54].

**Figure 1:**
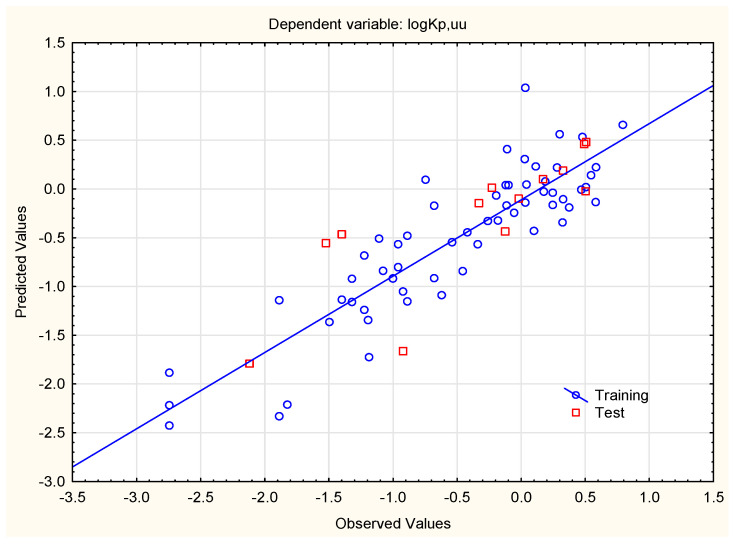
Predicted (Equation (4)) vs. observed (experimental) values of log ***K***_p,uu,br_ (rat reference data used in this model are considered to be in sufficient agreement with human data to be used in pre-clinical screening of drugs as CNS active/inactive [59,60]).

Until the early 2000s ***BB*** was the major parameter describing the compounds’ ability to enter the brain, with over 1000 compounds whose ***BB*** values are available [55]—and it is still studied (e.g., [51]) and calculated to assess the solutes’ brain uptake, using online platforms such as pkCMS or preADMET (https://preadmet.qsarhub.com/adme/, accessed on 10 November 2024). However, this parameter has some drawbacks—it is based on the total concentrations of solutes in blood and brain, whereas ***K***_p,uu_ accounts for binding to plasma proteins and brain tissues—this being an important development, since it is generally accepted that only unbound molecules interact with pharmacological targets in the brain. In this study the log ***BB*** and log ***PS*** values of 37 studied steroids are reported (as calculated using pkCMS software); the unbound parameters (log ***K***_p,uu,br_) were computed using a newly developed linear regression model (4) based on the set of compounds given by Lawrenz et al. [13] and four molecular descriptors (Figure 1).

log ***K***_p,uu,br_ = 4.909 (±1.372) − 0.00857 (±0.00079) ***W*** + 0.642 (±0.105) ***iLOGP*** + 0.893 (±0.297) ***MDCK*** − 0.393 (±0.184) ***PAMPA*** (4)

(n = 60, R^2^ = 0.783, R^2^_adj._ = 0.767, F = 49.54, *p* < 0.01, RMSEP_ext_ = 0.462, RMSECV = 0.425).

Further analysis of Equation (4) reveals that log ***K***_p,uu,br_ is positively correlated with compounds’ lipophilicity (***iLOGP***) and ***MDCK*** permeability—and negatively with molecules’ size (***MW***) and ***PAMPA*** permeability. Both MDCK (Madin–Darby canine kidney) cell lines [56] and parallel artificial membrane permeability assays (PAMPA) [57] are used to predict compounds’ membrane permeability, but they differ in respect to the mechanisms responsible for compounds’ passage, and the results obtained on them are not linearly correlated [58]. The analysis of coefficients in Equation (4) leads to conclusions which are to some degree contradictory with respect to the report by Friden et al. [59], who suggested that excessive ability to form H-bonds facilitates compounds’ efflux (for example by transporters such as P-gp). However, they did not observe the effect of lipophilicity upon log ***K***_p,uu,br_ (in contrast to log ***BB***, which is generally known to be lipophilicity-dependent).

**Figure 2:**
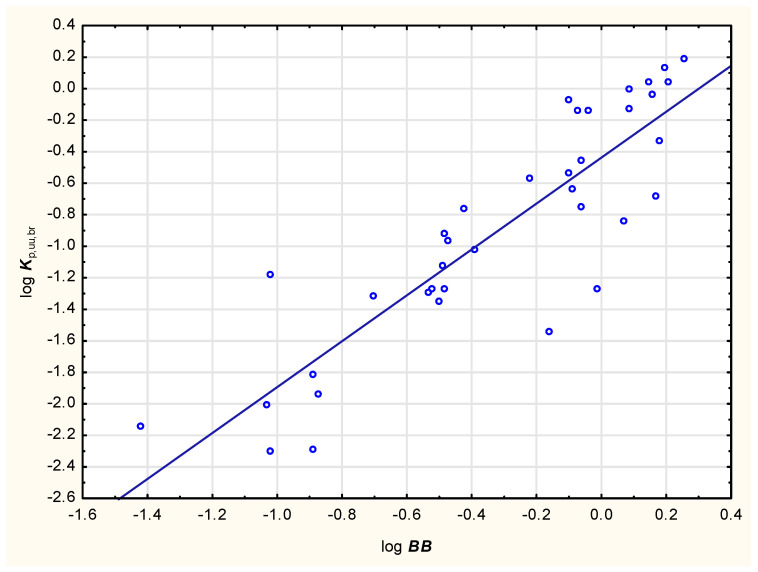
Relationship between log ***K***_p,uu,br_ (from Equation (4)) and log ***BB*** (calculated using pkCSM) for 37 steroids.

Values of log ***K***_p,uu,br_ calculated using Equation (4) are given in Table 1. Based on these values, studied steroids were classified as good or poor blood–brain barrier penetrators (BBB+ or BBB−, respectively). The ability of small molecules to reach therapeutic targets in the central nervous system (CNS) is related to their high unbound brain/plasma ratio (with often assumed cut-off values of ***K***_p,uu,br_ ≥ 0.3 to 0.5) [53]. As human ***K***_p,uu,br_ values are not easily accessible, data from animal models (mainly rats) are used. Friden and Winiwarter reported that “The structure-brain exposure relationships found in the rat also hold for humans, since the rank order of the drugs was similar for human and rat *K*_p,uu,CSF_” [59]. In lieu of human ***K***_p,uu,br_ data, the cut-off value (***K***_p,uu,br_ > 0.3) obtained on the rat model is accepted to select compounds with good CNS availability [60]. The results of the BBB+/− classification presented in this study are confirmed by earlier reports of poor blood–brain barrier permeability for dexamethasone, prednisolone or hydrocortisone—and good BBB permeability of testosterone [61,62]. Log ***K***_p,uu,br_ values calculated according to Equation (4) are in relatively good agreement (R^2^ = 0.77) with the computed log ***BB*** values (Figure 2).

### 3.7. Affinity of Steroid Drugs for the GST and NAT2 Enzymes

The GST and NAT2 enzymes in the placenta take part in deactivation of undesired xenobiotics and protect the fetus—but they can also play a less beneficial role, as they deactivate ant-cancer drugs in tumor cells, causing drug resistance to develop. The phenomenon of inhibiting the GST and NAT2 enzymes in order to prevent the enzymatic deactivation of drugs has been studied experimentally for large groups of GST and NAT inhibitors (registered or designed drugs) [63,64,65,66]. There is strong evidence that the activity of compounds as the GST and NAT2 enzyme inhibitors determined experimentally is related to their calculated affinity for the GST and NAT2 enzymes [67].

The binding affinities of selected steroids for GST and NAT2 enzymes were calculated by molecular docking methodology, as described in Section 2.4, and are presented in Table 3, along with the values obtained for reference ligands (glutathione for GST and coenzyme A for NAT2, respectively [18]).

It was established that all the studied steroids exhibit very high affinity for the GST enzyme, and that this affinity is particularly high for eplerenone. The affinities of steroid drugs for NAT2 are also high, compared to that of the reference ligand—with the exception of eplerenone and spironolactone, for which they are fairly similar to that for the reference ligand; binding affinities of studied steroids for both proteins are not interrelated (R^2^ = 0.11).

**Figure 3:**
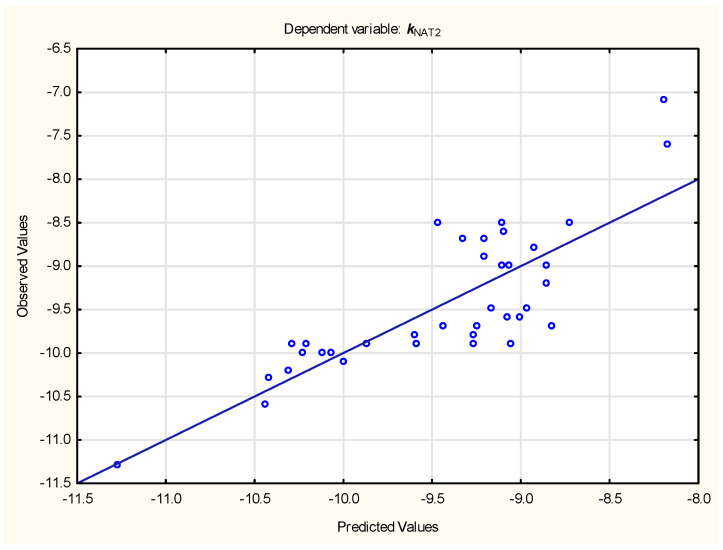
Predicted vs. observed ***k***_NAT2_ values—Equation (5).

**Figure 4:**
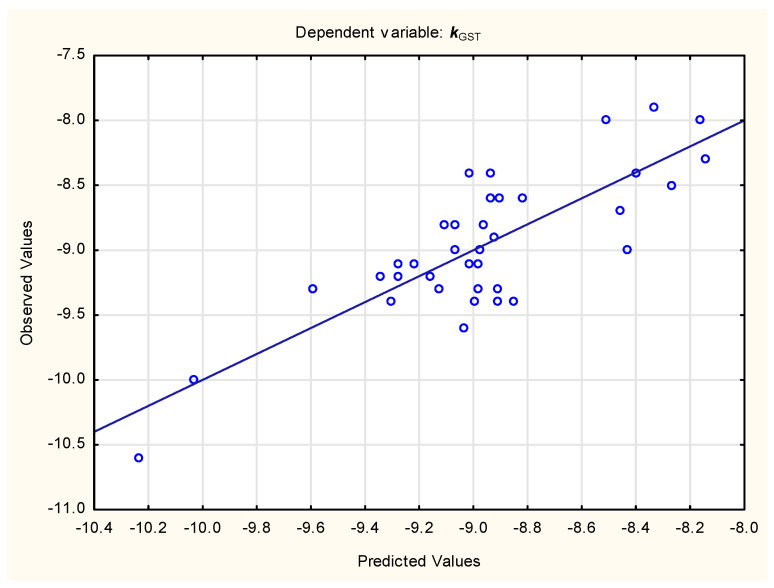
Predicted vs. observed ***k***_GST_ values—Equation (6).

At this point the relationships between steroid ligands’ affinity for studied proteins and the simple, calculated physico-chemical properties of ligands were investigated. Multiple linear regression (forward stepwise modes) led to the following Equations (5) and (6) (Figure 3 and Figure 4, respectively):

***k***_NAT2_ = −12.09 (± 1.05) − 0.207 (± 0.034) ***#Aromatic heavy atoms*** − 0.363 (± 0.072) ***#ROTATABLE_BONDS*** + 0.0242 (± 0.0075) ***SURFACE_AREA*** (5)

(n = 37, R^2^ = 0.665, R^2^_adj_. = 0.634, F = 21.81, *p* < 0.01, s_e_ = 0.49)

***k***_GST_ = −5.170 (± 0.883) + 0.326 (± 0.053) ***#ROTATABLE_BONDS*** − 0.0296 (± 0.059) ***SURFACE_AREA*** + 0.0694 (± 0.0181) ***MW*** − (± 0.262) ***#Heavy atoms*** + 0.132 (± 0.050) ***LOGP*** (6)

(n = 37, R^2^ = 0.658, R^2^_adj_. = 0.603, F = 11.92, *p* < 0.01, s_e_ = 0.88).

The statistics of Equations (5) and (6) are not entirely satisfying; nevertheless, it may be observed that the affinity of studied steroids for both enzymes is linked to ligand properties such as rotatable-bond count (***#ROTATABLE_BONDS***) and surface area (***SURFACE_AREA***). Apart from these properties, the affinity for the NAT2 protein is related to aromatic heavy-atom count (***#Aromatic heavy atoms***), and for GST to molecular weight (***MW***), heavy-atom count (***#Heavy atoms***) and lipophilicity (***LOGP***), respectively (Table 4). Interestingly, the signs of coefficients for ***#ROTATABLE_BONDS*** and ***SURFACE_AREA*** in Equations (5) and (6) are different, which implies an opposite influence of both properties on ligands’ binding affinities for studied proteins.

### 3.8. Key Steroids That May Become an Issue

Steroids are used to treat a variety of medical conditions, or as appearance- and performance-enhancing drugs. In this study, it was established that steroid drugs (including those identical to naturally occurring hormones) may become a serious environmental concern, once released into the ecosystem. The majority of sample steroids analyzed in this study are mobile in the soil–water compartment (only 3 out of 37 compounds are expected to be hardly mobile or immobile), so they can travel far from the point of release, to the environment. They may be absorbed by aquatic organisms—but, luckily, in the majority of cases, they are unlikely to bioaccumulate. Steroid drugs discussed in our research exhibit an ability to cross biological barriers—they are well absorbed from the gastrointestinal tract and through skin; once absorbed by a body of a female in gestation, they are likely to cross the placenta (the majority of them very easily) and, at the same time, to interact with placental enzymatic mechanisms of defense against xenobiotics (such as enzymes—glutathione S-transferase and N-acetyltransferase). They have also a relatively high potential to enter the brain—over 30% of studied compounds are known or expected to cross the blood–brain barrier (BBB+). Out of this group, five compounds are likely to be substrates for brain-to-blood efflux systems, such as P-glycoprotein (P-gp)—this, however, does not necessarily imply that they are CNS-inactive [68].

The results of our in silico investigations of studied steroids suggest that there are 10 compounds that are especially likely to be an issue in the context of pregnancy (Table 5). Some of them are naturally occurring hormones, but they are also administered as drugs; the majority are relatively mobile in the soil–water compartment and bioconcentrate in aquatic organisms (although they are, generally speaking, not very bioaccumulative), so they are likely to emerge unexpectedly far from the site where they have originally been released. They can also be easily absorbed though skin or from the gastrointestinal tract, cross the blood–brain barrier (likely of both mother and baby), and the placenta, and interact with placental enzymes.

## 4. Conclusions

It is not easy to evaluate the influence of steroid drugs upon an unborn baby. Some steroids are used during pregnancy to treat conditions including rheumatoid arthritis, systemic lupus erythematosus and inflammatory bowel disease—and the knowledge of these drugs’ effects related to child’s health and development is still limited [69]. The results of steroids’ examination as the BBB penetrators presented in this study rely upon data collected for a mature rather than a developing brain—but one should not forget that the blood–brain barrier begins to protect child’s brain at early stages of gestation [70], and the protective mechanisms it provides vary during fetal development. Certain glucocorticoids are administered to pregnant women to prevent premature birth; these compounds (as it was proved in vitro) intensify the P-gp expression of a fetus, protecting its brain against undesired xenobiotics and regulating the uptake of cortisol and aldosterone that take part in normal brain development [70]. Steroid drugs’ ability to circulate in the environment and to cross biological barriers in (sometimes) unaware individuals can make these compounds a health issue—but there are still a lot of gaps in our understanding of steroid drugs’ role, especially in developing organisms.

## Figures and Tables

**Table 1:** Calculated biological properties of 37 steroid drugs.

Compound	Log *K*_oc_	Mobility ^1^	Log *BCF* ^2^	Log *BB* ^3^	Log *PS* ^3^	Log *K*_p,uu,br_	BBB+/− ^4,5^	Pgp Substrate ^3^	*HIA*% ^3^	log *K*_p_ ^3^	log *FM* ^6^	Lipinski Violations ^7^
Dexamethasone	2.02	MM/SM	0.95	−0.70	−3.42	−1.32	BBB−	Yes	81.31	−3.97	−0.33	0
Hydrocortisone (HC)	1.72	**VM/M**	0.73	−0.48	−2.59	−1.27	BBB−	Yes	75.27	−4.35	−0.43	0
Progesterone	3.73	MM/SM	2.22	0.09	−1.93	−0.01	BBB+	No	99.27	−2.22	−0.46	0
Prednisolone	1.72	**VM/M**	0.74	−0.50	−3.36	−1.36	BBB−	Yes	73.69	−4.47	−0.40	0
Estrone	3.70	MM/SM	1.73	−0.04	−1.74	−0.14	**BBB+**	**Yes**	96.42	−2.62	−0.41	0
Aldosterone	0.77	**VM/M**	0.50	−0.16	−2.55	−1.55	BBB−	Yes	79.71	−4.45	−0.42	0
Corticosterone	1.71	**VM/M**	0.95	0.07	−2.22	−0.85	BBB−	Yes	96.95	−4.29	−0.48	0
Pregnenolone	3.43	MM/SM	2.45	0.09	−2.37	−0.13	BBB+	No	95.63	−2.77	−0.52	0
17-α-Hydroxyprogesterone	2.77	MM/SM	1.76	0.18	−1.75	−0.33	**BBB+**	**Yes**	96.03	−3.40	−0.47	0
17-α-Hydroxypregnenolone	2.55	MM/SM	2.17	−0.09	−1.70	−0.64	BBB−	Yes	95.73	−3.17	**−0.61**	0
Deoxycorticosterone	2.73	MM/SM	1.57	−0.06	−1.90	−0.46	**BBB+**	**Yes**	98.19	−3.41	−0.47	0
Testosterone	2.94	MM/SM	1.86	0.16	−2.08	−0.04	BBB+	No	96.54	−3.06	−0.45	0
Cortexolone	2.02	MM/SM	1.70	0.17	−2.26	−0.69	BBB−	Yes	96.58	−4.18	−0.49	0
Estradiol	3.54	MM/SM	2.31	−0.07	−1.33	−0.14	**BBB+**	**Yes**	93.90	−2.97	−0.43	0
Estriol	2.35	MM/SM	1.28	−0.22	−2.10	−0.57	BBB−	Yes	94.47	−3.29	−0.43	0
Cortisone	1.68	**VM/M**	0.64	−0.01	−2.50	−1.27	BBB−	Yes	88.79	−4.71	−0.41	0
HC succinamate	1.32	**VM/M**	0.61	−0.89	−3.34	−2.29	BBB−	Yes	71.11	−3.60	−0.46	0
HC N,N-dimethylsuccinamate	1.49	**VM/M**	1.01	−0.89	−3.16	−1.82	BBB−	Yes	74.44	−3.76	−0.45	0
HC methylsuccinate	1.62	**VM/M**	1.38	−0.49	−2.82	−1.13	BBB−	Yes	77.28	−3.45	**−0.53**	0
HC hemisuccinate	0.91	**VM/M**	0.50	−1.02	−3.29	−2.31	BBB−	Yes	50.08	−2.74	−0.48	0
HC pimelate	1.53	**VM/M**	0.50	−1.42	−2.86	−2.15	BBB−	Yes	53.29	−2.74	−0.42	1
HC pimelamate	1.95	**VM/M**	1.18	−1.03	−3.17	−2.01	BBB−	Yes	72.20	−3.41	−0.41	1
HC 6-hydroxyhexanoate	2.38	MM/SM	1.51	−1.02	−3.12	−1.19	BBB−	Yes	77.50	−3.71	−0.51	0
HC propionate	1.86	VM/M	1.51	−0.42	−2.79	−0.77	BBB−	Yes	83.46	−4.28	−0.43	0
HC methylpimelate	2.31	MM/SM	2.11	−0.52	−2.99	−1.27	BBB−	Yes	74.62	−3.27	−0.48	1
HC hexanoate	2.71	MM/SM	2.62	−0.48	−2.73	−0.92	BBB−	Yes	85.63	−3.83	**−0.57**	0
HC octanoate	3.25	MM/SM	**3.29**	−0.53	−2.70	−1.30	BBB−	Yes	87.03	−3.47	**−0.63**	0
Estradiol benzoate	4.26	HM/IM	**3.28**	−0.10	−1.31	−0.08	BBB+	No	97.41	−2.67	−0.49	1
HC acetate	1.29	**VM/M**	0.50	−0.87	−3.22	−1.94	BBB−	Yes	77.35	−3.36	−0.37	0
Deoxycortisone acetate	3.32	MM/SM	1.70	−0.06	−1.70	−0.76	BBB−	No	100.00	−2.57	−0.49	0
Cortisone acetate	2.39	MM/SM	1.05	−0.39	−2.66	−1.03	BBB−	Yes	86.28	−4.27	−0.41	0
Testosterone propionate	4.03	HM/IM	2.81	0.26	−2.38	0.19	BBB+	No	97.44	−2.93	−0.49	0
Methyltestosterone	3.04	MM/SM	1.88	0.21	−2.41	0.04	BBB+	No	95.58	−2.95	**−0.55**	0
Testosterone enanthate	5.09	HM/IM	**4.11**	0.20	−2.08	0.13	BBB+	No	95.00	−2.78	**−0.55**	1
Spironolactone	2.89	MM/SM	1.16	−0.10	−1.56	−0.54	BBB−	No	97.92	−3.35	−0.51	0
Eplerenone	2.58	MM/SM	0.69	−0.47	−2.95	−0.97	BBB−	No	100.00	−3.23	−0.46	0
Tibolone	3.36	MM/SM	2.26	0.15	−2.46	0.04	**BBB+**	**Yes**	96.26	−3.11	−0.51	0

^1^ Mobility in soil according to EPA: very mobile/mobile (VM/M); moderately mobile/slightly mobile (MM/SM), hardly mobile/immobile (HM/IM)—very mobile/mobile compounds are highlighted (bold); ^2^ compounds expected to bioaccumulate in aquatic organisms are highlighted (bold); ^3^ calculated using pkCSM; ^4^ BBB+ compounds that are also suspected P-gp substrate are highlighted in bold; ^5^ compounds that are likely to cross the placenta and BBB are underlined; ^6^ Compounds formally non-crossing the placenta are highlighted in bold; ^7^ calculated using SwissADME.

**Table 2:** Mobility classification according to EPA.

Range of Log *K*_oc_	Mobility Class
<1	Very mobile
1–2	Mobile
2–3	Moderately mobile
3–4	Slightly mobile
4–5	Hardly mobile
>5	Immobile

**Table 3:** Affinities of steroid drugs for GST and NAT2 ligands (kcal/mol).

	*k*_GST_	*k*_NAT2_
Dexamethasone	−9.1	−9.0
Hydrocortisone (HC)	−9.3	−8.6
Progesterone	−9.6	−9.6
Prednisolone	−9.2	−8.5
Estrone	−9.1	−10.6
Aldosterone	−9.4	−8.5
Corticosterone	−9.4	−8.7
Pregnenolone	−8.4	−9.0
17-α-Hydroxyprogesterone	−9.2	−9.5
17-α-Hydroxypregnenolone	−8.6	−8.8
Deoxycorticosterone	−9.4	−8.7
Testosterone	−8.8	−9.6
Cortexolone	−9.3	−8.9
Estradiol	−8.9	−10.3
Estriol	−9.0	−10.2
Cortisone	−9.1	−9.0
HC succinamate	−9.1	−9.9
HC N,N-dimethylsuccinamate	−9.4	−9.8
HC methylsuccinate	−8.4	−9.7
HC hemisuccinate	−8.6	−9.8
HC pimelate	−8.5	−10.0
HC pimelamate	−9.0	−9.9
HC 6-hydroxyhexanoate	−7.9	−10.0
HC propionate	−8.8	−9.7
HC methylpimelate	−8.7	−10.0
HC hexanoate	−8.0	−9.9
HC octanoate	−8.3	−9.9
Estradiol benzoate	−10.0	−11.3
HC acetate	−9.3	−9.7
Deoxycortisone acetate	−9.0	−9.9
Cortisone acetate	−9.2	−9.9
Testosterone propionate	−8.6	−9.5
Methyltestosterone	−8.8	−9.2
Testosterone enanthate	−8.0	−10.1
Spironolactone	−9.3	−7.6
Eplerenone	−10.6	−7.1
Tibolone	−8.4	−8.5
Reference ^1^	−5.4	−7.4

^1^ Reference ligands: Glutathione for GST, CoA for NAT2 enzymes.

**Table 4:** Order of selection in forward stepwise regression, signs of coefficients and % of total variability accounted for by particular descriptors.

	GST			NAT2		
**No.**	**Descriptor ^1^**	**Sign ^1^**	**%**	**Descriptor ^2^**	**Sign ^2^**	**%**
1	***#ROTATABLE_BONDS***	**+**	19	***#Aromatic heavy atoms***	**−**	28
2	***SURFACE_AREA***	**−**	20	***#ROTATABLE_BONDS***	**−**	28
3	***MW***	**+**	7.5	***SURFACE_AREA***	**+**	10.5
4	***#Heavy atoms***	**−**	11.5			
5	***LOGP***	**+**	8			

^1^ Equation (6); ^2^ Equation (5).

**Table 5:** Summary of studied properties of steroids that can cross both the blood–brain barrier and placenta.

Compound	Soil Mobility ^1^	Bioaccumulation ^2^	GI Absorption ^3^	Skin Permeability ^4^	Placenta Permeability ^5^	CNS Availability ^6^	GST Affinity ^7^	NAT2 Affinity ^8^
Progesterone	Medium	Low	High	Yes	Yes	Yes	High	High
Estrone	Medium	Low	High	Yes	Yes	Yes	High	High
Pregnenolone	Medium	Low	High	Yes	Yes	Yes	High	High
17-α-OH-progesterone	Medium	Low	High	Yes	Yes	Yes	High	High
Deoxycorticosterone	Medium	Low	High	Yes	Yes	Yes	High	High
Testosterone	Medium	Low	High	Yes	Yes	Yes	High	High
Estradiol	Medium	Low	High	Yes	Yes	Yes	High	High
Estradiol benzoate	Low	High	High	Yes	Yes	Yes	High	High
Testosterone propionate	Low	Low	High	Yes	Yes	Yes	High	High
Tibolone	Medium	Low	High	Yes	Yes	Yes	High	High

^1^ Medium soil mobility—log ***K***_oc_ 2 to 4; low soil mobility—log ***K***_oc_ > 4; ^2^ low bioaccumulation—log ***BCF*** < 3; ^3^ according to pkCSM (molecules with HIA absorption below 30% are considered poorly absorbed); ^4^ based on pkCSM; ^5^ placenta permeability classification based on calculated (Equation (3)) ***FM*** > 0.3 cut-off value; ^6^ CNS availability “Yes”—rat ***K***_p,uu,br_ > 0.3 calculated according to Equation (4); ^7^ ***k***_GST_ more negative than that of glutathione; ^8^ ***k***_NAT2_ more negative than that of CoA.

## Data Availability

The original contributions presented in this study are included in the article/Supplementary Materials. Further inquiries can be directed to the corresponding authors.

## Supplementary Materials

The following supporting information can be downloaded at: https://www.mdpi.com/article/10.3390/membranes15010004/s1, File S1: Compounds’ Mordred, pkCSM and Mordred molecular descriptors; descriptors’ co-linearity (tolerances).
